# The causality between gut microbiome and anorexia nervosa: a Mendelian randomization analysis

**DOI:** 10.3389/fmicb.2023.1290246

**Published:** 2023-10-19

**Authors:** Xuan Xia, Shu-yang He, Xiao-Lin Zhang, Decheng Wang, Qian He, Qing-Ao Xiao, Yong Yang

**Affiliations:** ^1^Hubei Key Laboratory of Tumor Microenvironment and Immunotherapy, College of Basic Medical Science, China Three Gorges University, Yichang, China; ^2^Institute of Infection and Inflammation, China Three Gorges University, Yichang, China; ^3^Department of Physiology and Pathophysiology, College of Basic Medical Science, China Three Gorges University, Yichang, China; ^4^Department of Critical Care Medicine, Yiling People’s Hospital of Yichang City, Yichang, China; ^5^Department of Interventional Radiology, The First College of Clinical Medical Science, China Three Gorges University, Yichang, China; ^6^Yichang Central People’s Hospital, Yichang, China; ^7^Hunan Provincial Key Laboratory of Metabolic Bone Diseases, Department of Metabolism and Endocrinology, National Clinical Research Center for Metabolic Diseases, The Second Xiangya Hospital of Central South University, Changsha, China

**Keywords:** gut microbiome, Mendelian randomization, anorexia nervosa, genome-wide association study, causality

## Abstract

**Background and aim:**

Nutrient production by intestinal microbiota corresponds to regulate appetite while gut microbial composition was influenced by diet ingestion. However, the causal relationship between gut microbial taxa and anorexia nervosa (AN) remains unclear. Mendelian Randomization (MR) is a novel research method that effectively eliminates the interference of confounding factors and allows for the exploration of the direct causal effects between exposure and outcome. This study employs MR to explore the causal effect between AN and specific gut microbiome.

**Methods:**

Large-scale Genome Wide Association Study (GWAS) data of AN and 211 gut microbes were obtained from the IEU open GWAS project and Mibiogen Consortium. Two-sample MR was performed to determine the causal relationship between gut microbiota and AN. Furthermore, a bi-directional MR analysis was to examine the direction of the causal relations. The Bonferroni correction test was used to adjust potential correlations among microbial taxa.

**Result:**

In forward MR analysis, 10specific gut microbial taxa have an impact on the occurrence of AN (the *p* value of IVW <0.05). The high abundance of *Genus Eubacteriumnodatumgroup ID: 11297* (OR:0.78, 95% CI:0.62–0.98, *p* = 0.035) and *Class Melainabacteria ID: 1589* (OR:0.72, 95% CI:0.51–0.99, *p* = 0.045) may be considered protective factors for AN. But after Bonferroni correction, only *Class Actinobacteria ID:419* (OR:1.53, 95% CI:1.19–1.96, *p* = 0.00089) remained significantly associated and high abundance of *Class Actinobacteria ID:419* considered as a risk factor for AN. In the reverse MR analysis, AN influences 8 gut microbial taxa with none-statistically significant associations after adjustment.

**Conclusion:**

We identified a significant correlation between AN and 18 microbial taxa which have not been previously reported. Among them, 10 kinds of gut bacteria may affect the occurrence of AN, and the status of AN would affect 8 kinds of gut bacteria. After correction, the *Class Actinobacteria ID:419* continued to exert an influence on AN.

## Graphical abstract

The left half of the figure shows the forward Mendelian randomization results showing the causal effect of gut microbiota on AN. The right half of the figure shows the reverse Mendelian randomization results showing the causal effect of AN on the gut microbiota. Risk factors are indicated in red font and protective factors in dark green font.

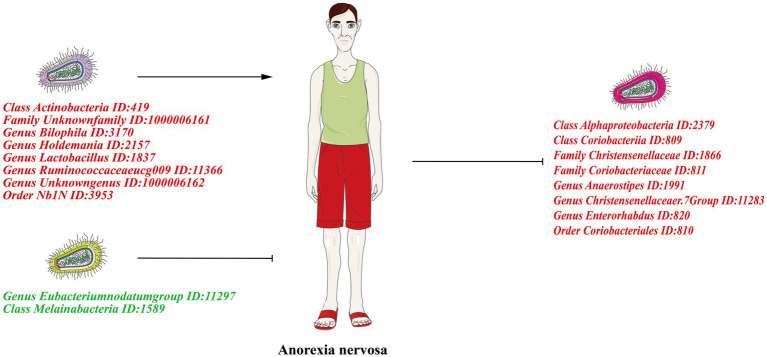

## 1. Introduction

Anorexia nervosa (AN) is one of the most serious psychiatric disorders, characterized by life-threatening very-low body weight, nutritional restrictions, and obviously occurs in females and adolescents (Treasure et al., 2015; Bulik et al., 2019; Seitz et al., 2019). Its prevalence in the population is estimated to be around 1% (Fan et al., 2023). Over the past decade, there has been a progressively increasing trend in the incidence of anorexia nervosa. This rising incidence has been observed in several countries such as the United Kingdom, Germany, and Japan (Holland et al., 2016; Bulik et al., 2019). The aggregate mortality is estimated to approximately 5.6% per decade, significantly surpassing that of the general population (Mitchell et al., 2020). However, AN suffers from efficient therapeutic methods and liability to relapse (Bulik et al., 2019). Previously researches displayed the incidence of AN accompanied by multiple dysfunctions, including endocrine alterations (Schorr and Miller, 2017), increased inflammation (Dai et al., 2013), and immune response (Seitz et al., 2019).

The human gastrointestinal tract harbors a multitude of microorganisms. Previous research has indicated that any disruption in the composition of the gut microbiota can lead to the onset of liver diseases (Tilg et al., 2022). These microorganisms can modulate individual behavior through various mechanisms, including metabolite, endocrine pathways (Albillos et al., 2020). The gut microbiota’s involvement in the development of neurological disorders through the microbiota-gut-brain axis has been reported for some time (Schorr and Miller, 2017; Mossad et al., 2021; Drljaca et al., 2023). However, the specific role played by individual microbial groups in this process remains unclear. Recently, the crucial role of the gut microbiome in AN has been gradually unveiled. Nevertheless, exploring the connection between AN and the gut microbiota poses challenges owing to the susceptibility to various influencing factors. A recent meta-analysis has unveiled links between AN and numerous microbial taxa. However, numerous studies have generated conflicting outcomes regarding the precise relationships between particular microbial taxa and AN (Nikolova et al., 2021). The reasons for these divergent results are multifaceted. Firstly, there’s the influence of confounding factors like environmental elements (such as mental status, sleep status, etc.). Secondly, the intricate interplay among gut microbiota complicates the causal effect between individual microbial groups and AN, making it difficult to reveal cause-and-effect relationship. Currently, there is also a lack of large-scale randomized controlled trials (RCTs) to substantiate this causal link.

Mendelian randomization (MR) is a genetic epidemiological approach that utilizes instrumental variables (IVs) which are highly correlated with the exposure of interest, to investigate causality and mitigate the impact of confounding factors (Emdin et al., 2017; Davies et al., 2018). This method capitalizes on the inherent property of Single Nucleotide Polymorphisms (SNPs) to be randomly assorted and distributed during gamete formation, rendering them unaffected by confounding factors following gametogenesis. Furthermore, the non-reversible nature of heredity helps in excluding the possibility of reverse causal effects (Bowden et al., 2015). In essence, MR, as an innovative methodology, offers the means to address confounding and reverse causality by leveraging genetic variants with close associations to the exposure of interest in order to infer potential causal relationships with the outcome (Emdin et al., 2017; Davies et al., 2018).

In this study, we investigated the potential causal effect between 211 gut microbiota taxa and AN by MR study design. After correction, the *Class Actinobacteria ID:419* was found to exert an influence on AN. Our study provides a new sight into exploring the relationship between AN and gut microbiota.

## 2. Materials and methods

### 2.1. Study design

**Figure 1:**
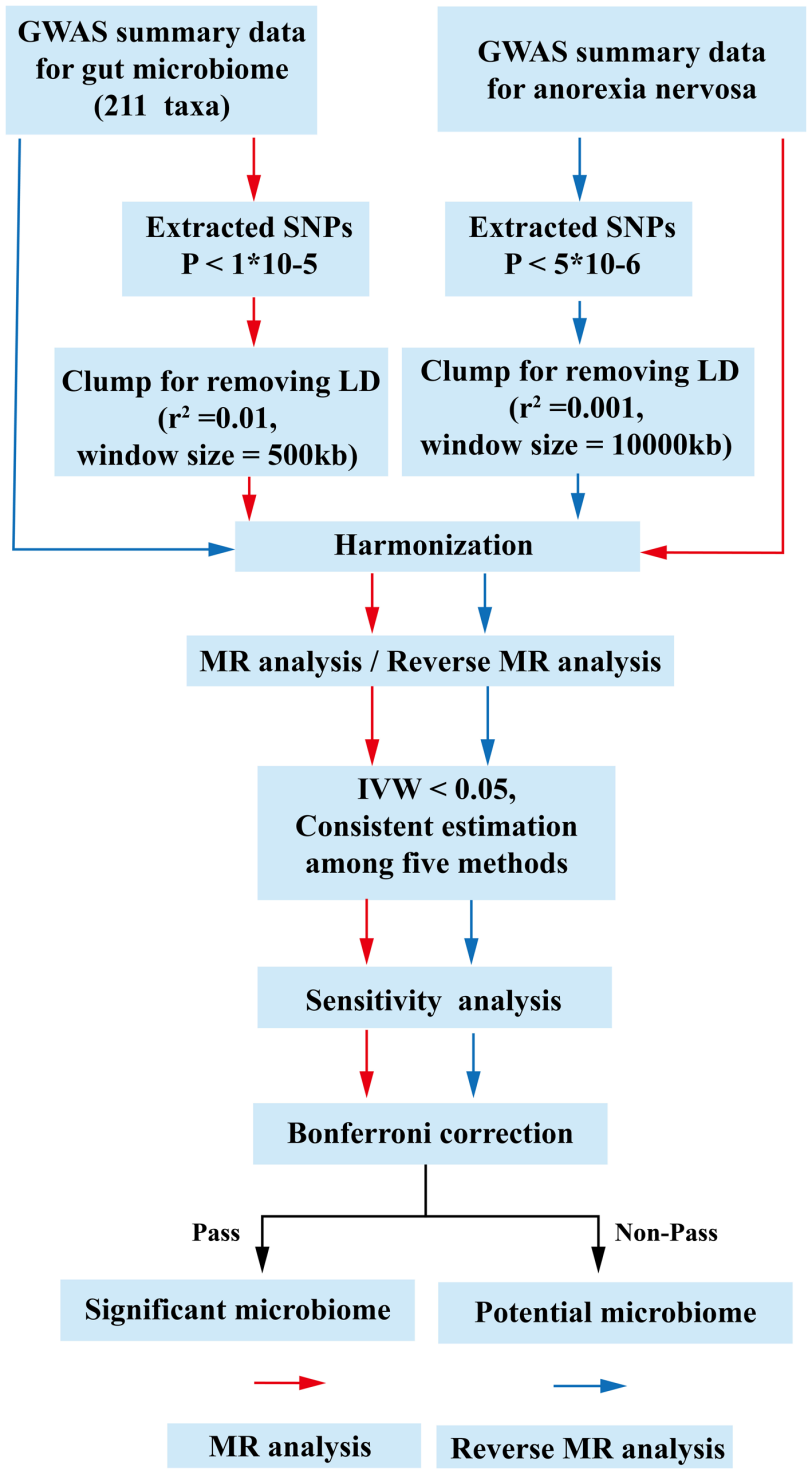
The flow chart of the study. The GWAS data of flora was used as exposure, and anorexia nervosa was used as outcomes for MR analysis. The instrumental variables of flora were extracted in the following way: (1) *p* < 1 × 10^−5^; (2) *r*^2^ = 0.01, kb = 500. Five methods were used for MR analysis after harmonization. Floras with IVW estimates below 0.05, exhibiting consistent results across the five analytical methods, were deemed to hold potential association. Subsequently, an investigation into the presence of pleiotropy and heterogeneity among these potential floras was undertaken. Those floras displaying pleiotropic or heterogeneous characteristics were promptly eliminated. Meaningful bacterial groups were screened out. Then reverse MR analysis was performed, and different criteria were used for the screening of instrumental variables for AN (*p <* 5 × 10^−6^, *r*^2^ = 0.001, kb = 10,000). All potential floras were corrected by the Bonferronitest. The floras passed the Bonferroni were considered as significant. MR, Mendelian randomization; LD, linkage disequilibrium; GWAS, Genome Wide Association Study; SNPs, single nucleotide polymorphisms; IVW, inverse variance weighting.

Two hundred and eleven gut microbiota taxa were selected as exposure and AN was defined as outcome for MR analysis. Then, the exposure and outcome were exchanged for reverse MR analysis. All MR analysis of this study was executed under three basic assumptions: (1) IVs must be strongly correlated with exposure; (2) IVs cannot be correlated with confounding factors; (3) IVs can only affect outcomes through exposure factors (Bowden and Holmes, 2019). The GWAS data we selected all originate from populations of European ancestry. These individuals are largely independent of each other. The flow-chart of this study was shown in Figure 1. Furthermore, we used the STROBE-MR (Strengthening the Reporting of Observational Studies in Epidemiology-Mendelian Randomization) checklist to explain our MR study (Skrivankova et al., 2021). The checklist shown in Supplementary Table S1.

### 2.2. GWAS data of gut microbiome

The large-scale GWAS summary data of gut microbiome were obtained from Mibiogen consortium, including 18,340 individuals from 24 cohorts (Kurilshikov et al., 2021). The consortium utilized standardized analytical pipelines for both microbiota phenotype and genotype, ensuring uniform data processing methods. This approach was employed to mitigate potential variations introduced by technical differences in generating microbiota data. This study used three different regions (V4: 10,413 samples, 13 cohorts, V3-V4: 4,211 samples, 6 cohorts and V1-V2: 3,716 samples, 5 cohorts) (V: hypervariable region sequencing for identifying bacterial taxa)of the 16S rRNA gene to analysis the composition of gut microbiota and identified genetic variants that influent the relative abundance of microbial taxa by use of microbiota Quantitative Trait loci (mbQTL) mapping (Kurilshikov et al., 2021). In the original study, the gut microbiota was categorized into 257 taxa at five taxonomic levels: Phylum, Class, Order, Family, and Genus. Finally, 211 taxa were defined, including 131 genera, 35 families, 20 orders, 16 classes, and 9 phyla. All data from this study are publicly accessible, which could acquire from the website (https://mibiogen.gcc.rug.nl/menu/main/home/).

### 2.3. GWAS data of AN

The GWAS summary data of AN were obtained from the IEU open GWAS project (v7.5.5-2023-08-09, *N* = 42,348, https://gwas.mrcieu.ac.uk/). The GWAS summary dataset encompassed 2,907 individuals diagnosed with AN across 14 countries, along with 14,860 ancestrally matched control subjects, constituting a part of the Genetic Consortium for Anorexia Nervosa (GCAN) and the Wellcome Trust Case Control Consortium 3 (WTCCC3) (Boraska et al., 2014). All individuals were of European ancestry and detailed information of ANwas available in the website (https://gwas.mrcieu.ac.uk/datasets/ieu-a-45/).

### 2.4. The selection of IVs

The criterion of selecting IVs as following: (1) SNPs, significantly associated with gut microbiota, were selected (the *p* value of SNPs <1 × 10^−5^) as the potential eligible IVs (Yu et al., 2023); (2) SNPs were clumped for excluding the effect of linkage disequilibrium (*r*^2^ = 0.01, window size = 500 kb) (Xu et al., 2021); (3)palindromic alleles were removed. Then, For the reverse MR analysis, we filtered the IVs of AN. *p ≤* 5 × 10^−6^was chose as the criterion and clump were reset (*r*^2^ = 0.001, window size = 10,000 kb) (Yu et al., 2023). Other criterions were the same as above. To avoid weak instrumental bias, the F statistics for each bacterial taxon was calculated by following equation (Xu et al., 2021):


$$F=R^{2}\times \frac{n-1-k}{\left(1-R^{2}\right)\times k}$$

In this equation, *R*^2^ is to explain exposure variance of the IVs, *n* is the sample size, and k is the number of IVs (Xu et al., 2021). According to previous study, F statistic ≥10 were considered that there is no weak instrument bias (Pierce et al., 2011).

### 2.5. MR analysis

Five methods (including Inverse variance weighted (IVW), MR Egger, Weighted median, Simple mode, and Weighted mode method) were used to estimate the causal effect of the gut microbiota on AN. Due to its assumption that all instrumental variables are valid, IVW is susceptible to the effects of instrumental variable pleiotropy and heterogeneity (Bowden et al., 2016). However, in the absence of these influences, IVW considered to be the most accurate method, even when the other four methods may not yield positive results (Long et al., 2023).

To establish the accuracy and seriousness, five distinct methods was employed to assess the causal impact of gut microbiota on AN, namely: (1) Inverse Variance Weighted (IVW); (2) MR Egger; (3) Weighted Median (WM); (4) Simple Mode; (5) Weighted Mode method. It’s worth noting that, while the IVW method assumes the validity of all instrumental variables, it can be influenced by instrumental variable pleiotropy and heterogeneity. However, in scenarios devoid of these influences, IVW remains esteemed as the most precise technique (Wang et al., 2023). Thus, the result of MR analysis was mainly based on IVW (Burgess et al., 2013). The remaining four methods were considered supplementary to the IVW approach. Once instrumental variables (IVs) were harmonized, if the count of matched SNPs for a specific microbiota was <3, that microbiota was excluded due to lack of credibility in results.

A significance threshold for multiple testing was established at each taxonomic level (phylum, class, order, family, and genus). Bonferroni correction method was employed to adjust the *p*-values, mitigating the potential for false positives (*p* < 0.05/N, N refers to the effective number of independent bacterial taxa at the specific taxonomic level). The significant *p* values were following: 0.00038 (131 Genera), 0.0014 (35 Families), 0.0025 (20 Orders), 0.0031 (16 Classes), and 0.0056 (9 Phyla). Subsequently, when the estimated value of five methods exhibited congruence and IVW yielded a value <0.05/N, the gut microbes were deemed to exhibit a statistically significant distinction. Microbial taxa with the *p*-values of IVW below 0.05, although not statistically significant after Bonferroni correction, are considered to have potential associations.

The significant microbiotas were tested for pleiotropy and heterogeneity to ensure the accuracy of the IVW results. MR-Egger Intercept Test and Mendelian Randomization Pleiotropy RESidual Sum and Outlier (MR-PRESSO) global test were employed to detect horizontal pleiotropy (Long et al., 2023). MR-PRESSO could assess the overall horizontal pleiotropy of IVs and the abnormal SNPs which led to the pleiotropy (Verbanck et al., 2018). For assessing the presence of horizontal pleiotropy, *p* values greater than 0.05 for two methods were indicative of its absence. To gage the extent of heterogeneity, Cochran’s Q test was employed, with *p* values above 0.05 indicating non-heterogeneity. Therefore, in our study, any microbiota with Cochran’s Q test *p*-value remaining less than 0.05 were excluded from further analysis to ensure the reliable results of IVW method. Meanwhile, Leave-one-out analysis was employed to exclude the influence of single SNP.

### 2.6. Reverse MR analysis

To investigate whether AN exerted acausal influence on gut microbiomes, we additionally conducted a reverse MR analysis. In this procure, we employed SNPs strongly linked with AN as instrumental variables (IVs) to further probe the causal effect of AN on gut microbiome. The analytical approach was same with the MR analysis.

### 2.7. Data processing

All data processing and analysis were accomplished by R software (R.4.2.3; http://www.R-project.org). The R packages used in study is TwoSampleMR, MendelianRandomization, and MR-PRESSO.

## 3. Results

### 3.1. Instrumental variables for gut microbiome and AN

After strong correlation screening (*p <* 1 × 10^−5^) and clump (*r*^2^ = 0.01, window = 500 kb). SNPs associated with gut microbiota at difference levels were identified (Class: 231, Family: 509, Genus: 1740, Order: 289, Phylum: 126; Detail information shown in Supplementary Table S2). For AN, a threshold of 5 × 10^−6^ (*r*^2^ = 0.001, window = 10,000 kb) adopted for selection. Finally, 7 IVs were filtered and detail information was displayed in Supplementary Table S3.

### 3.2. Causal effects of gut microbiota on AN

**Figure 2:**
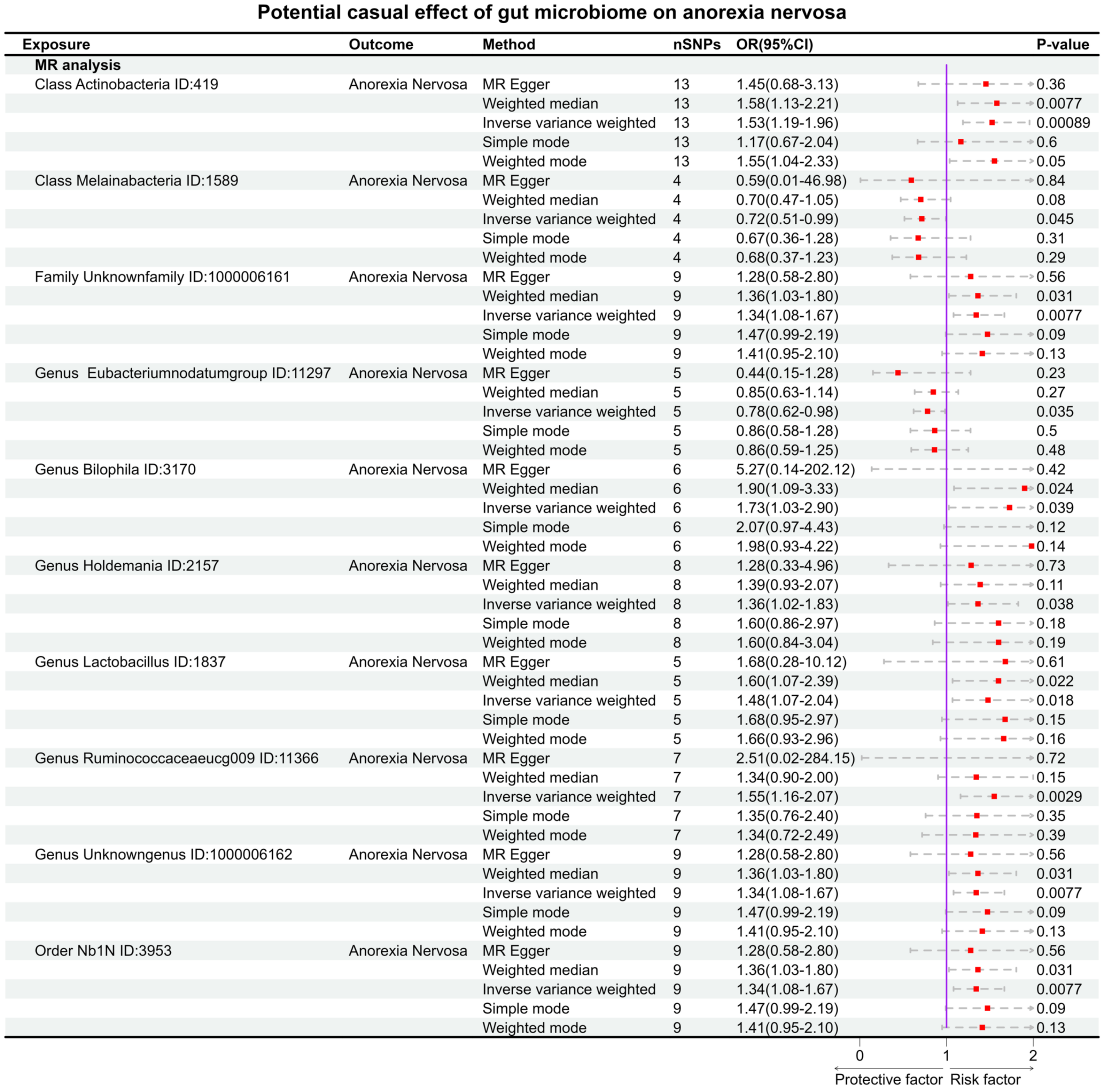
The result of MR between gut microbiome and anorexia nervosa by five methods.

**Figure 3:**
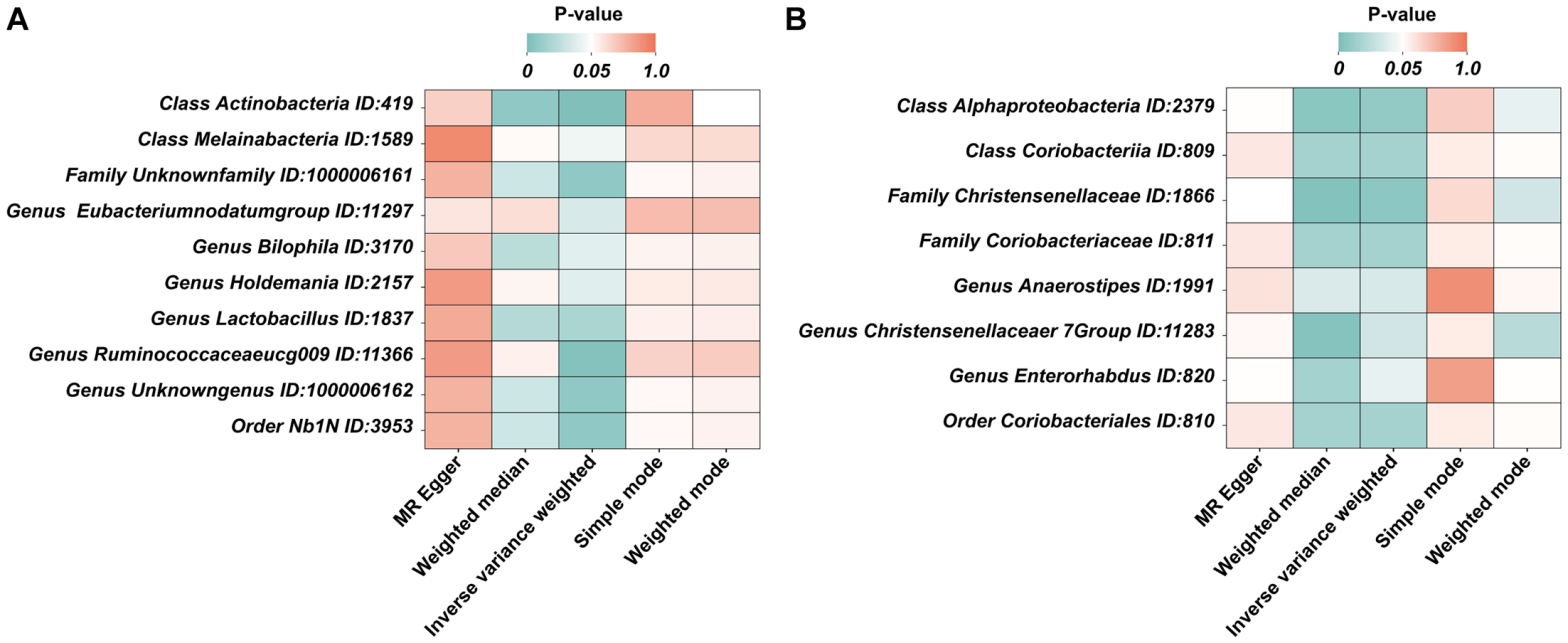
The heatmaps of five MR analysis methods. **(A)** The *p* value of five MR analysis (Forward); **(B)** The *p* value of five MR analysis (Reverse).

Under the condition of IVW < 0.05, 10 gut microbiotas were associated with AN (Figures 2, 3A). The result showed that high abundancy of 8 microbiotas were the risk factors for the onset of AN, including *Class Actinobacteria ID:419* (OR:1.53, 95% CI:1.19–1.96, *p* = 0.00089), *Family Unknown family ID:1000006161* (OR:1.34, 95% CI:1.08–1.67, *p* = 0.0077), *Genus Bilophila ID:3170* (OR:1.73, 95% CI:1.03–2.90, *p* = 0.039), *Genus Holdemania ID:2157* (OR:1.36, 95% CI:1.02–1.83, *p* = 0.038), *Genus Lactobacillus ID:1837* (OR:1.48, 95% CI:1.07–2.04, *p* = 0.018), *Genus Ruminococcaceaeucg009 ID:11366* (OR:1.55, 95% CI:1.16–2.07, *p* = 0.0029), *Genus Unknowngenus ID:1000006162* (OR:1.34, 95% CI:1.08–1.67, *p* = 0.0077), *Order Nb1N ID:3953* (OR:1.34, 95% CI:1.08–1.67, *p* = 0.0077). Two microbiotas were the protective factors for AN, including *Genus EubacteriumnodatumgroupID:11297*(OR:0.78, 95%CI:0.62–0.98, *p* = 0.035), *Class Melainabacteria ID:1589* (OR:0.72, 95% CI:0.51–0.99, *p* = 0.045). After Bonferroni correction, only one microbiota was significance associated with AN (*Class Actinobacteria ID:419*).

### 3.3. Causal effects of AN on gut microbiota

**Figure 4:**
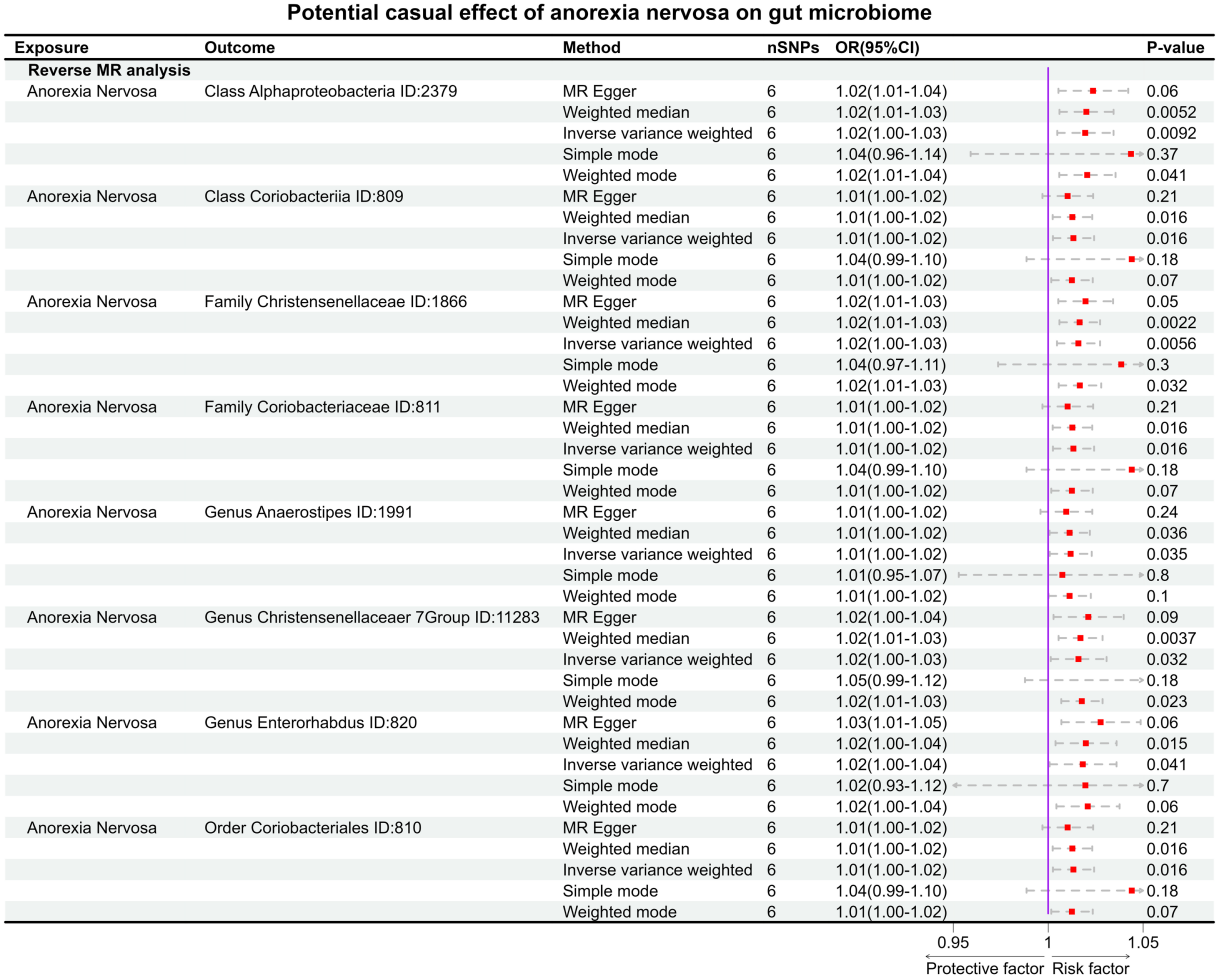
The result of reverse MR analysis between gut microbiome and anorexia nervosa by five methods.

In the reverse MR, the abundancy of eight gut microbial taxa were identified that are influenced by AN (Figures 3B, 4), including *Class Alphaproteobacteria ID:2379* (OR:1.02, 95% CI:1.00–1.03, *p* = 0.0092), *ClassCoriobacteriia ID:809* (OR:1.01, 95% CI:1.00–1.02, *p* = 0.016), *Family Christensenellaceae ID:1866* (OR:1.02, 95% CI:1.00–1.03, *p* = 0.0056), *Family Coriobacteriaceae ID:811* (OR:1.01, 95% CI:1.00–1.02, *p* = 0.016), *GenusAnaerostipesID:1991* (OR:1.01, 95% CI:1.00–1.02, *p* = 0.035), *Genus Christensenellaceaer.7Group ID:11283* (OR:1.02, 95% CI:1.00–1.03, *p* = 0.032), *Genus Enterorhabdus ID:820* (OR:1.02, 95% CI:1.00–1.04, *p* = 0.041), and *Order Coriobacteriales ID:810* (OR:1.01, 95% CI:1.00–1.02, *p* = 0.016). Under conditions of AN, these microbial taxa are suppressed and would display low abundancy. In essence, AN acted as a potential risk factor for these microbial communities. However, there was no significant microbiota after Bonferroni correction.

### 3.4. Sensitivity analysis of MR

**Figure 5:**
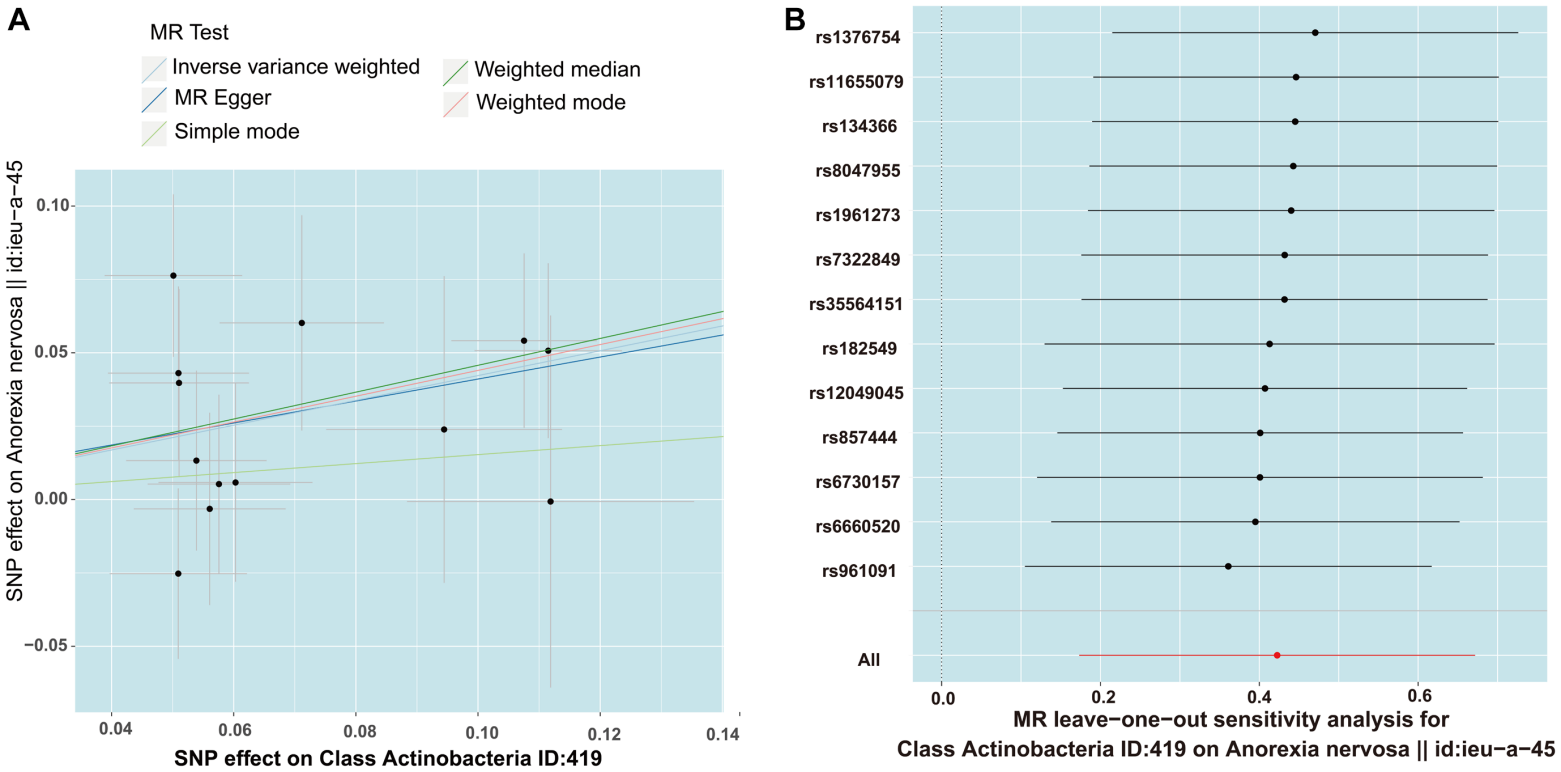
The result of MR between gut microbiome and anorexia nervosa by five methods. **(A)** In the scatter plot,estimated value of five MR tests were consistent. **(B)** The leave-one-out analysis validated that there is no significant differences in SNP effects between Class Actinobacteria ID:419 and anorexia nervosa.

MR PRESSO did not identify heterogeneity among the significant and potential microbiota (Tables 1, 2). Similarly, Cochran’s Q test indicated the absence of heterogeneity across the studies (Tables 1, 2). Moreover, the Leave-one-out analysis revealed no significant differences in SNP effects. Furthermore, the MR Egger regression did not yield any evidence of horizontal pleiotropy (*p* > 0.05), and all F-statistical values exceeded 10 (Tables 1, 2). The MR analysis and Leave-one out analysis result of *Class Actinobacteria ID:419* was shown in the Figure 5.

**Table 1:** The results of pleiotropy and heterogeneity in MR analysis.

Exposure	MR egger test	MR-PRESSO	Cochran’s Q test	*F* value
Class Actinobacteria ID:419	0.899	0.657	0.586	31.143
Class Melainabacteria ID:1589	0.941	0.537	0.486	22.372
Family Unknown family ID:1000006161	0.902	0.867	0.875	24.548
Genus eubacterium nodatum group ID:11297	0.359	0.798	0.749	21.083
Genus Bilophila ID:3170	0.577	0.221	0.167	21.236
Genus Holdemania ID:2157	0.932	0.523	0.475	22.753
Genus Lactobacillus ID:1837	0.897	0.763	0.733	22.170
Genus Ruminococcaceaeucg009 ID:11366	0.849	0.569	0.514	22.163
Genus Unknowngenus ID:1000006162	0.902	0.867	0.875	24.548
Order Nb1N ID:3953	0.902	0.867	0.875	24.548

The outcome of this MR analysis is anorexia nervosa.

**Table 2:** The results of pleiotropy and heterogeneity in MR analysis.

Outcome	MR egger test	MR-PRESSO	Cochran’s Q test	*F* value
Class Alphaproteobacteria ID:2379	0.495	0.567	0.573	1144.90
Class Coriobacteriia ID:809	0.491	0.592	0.766	1144.90
Family Christensenellaceae ID:1866	0.434	0.584	0.393	1144.90
Family Coriobacteriaceae ID:811	0.491	0.592	0.766	1144.90
Genus Anaerostipes ID:1991	0.600	0.687	0.898	1144.90
Genus Christensenellaceaer.7Group ID:11283	0.402	0.492	0.134	1144.90
Genus Enterorhabdus ID:820	0.205	0.486	0.354	1144.90
Order Coriobacteriales ID:810	0.491	0.592	0.766	1144.90

The exposure of this MR analysis is anorexia nervosa.

## 4. Discussion

As early as the 20th century, it was discovered that probiotics could improve human mental well-being, such as fatigue, melancholia and the neurosis (Bested et al., 2013). The composition of gut microbiota in individuals with mental illnesses significantly differs from that of healthy individuals. Preclinical experiments have demonstrated that transplanting fecal material from individuals with mental disorders into the intestines of germ-free mice can markedly alter the behavior in mice (Kelly et al., 2016; Zheng et al., 2016; Li et al., 2019; Sharon et al., 2019; Zhu et al., 2020; Fan et al., 2023). Furthermore, previous researches had already demonstrated the role of gut microbiota in regulating appetite (Breton et al., 2016; Fetissov, 2017; Han et al., 2021). This process may depend on tryptophan (Dong et al., 2020). Thus, some researchers are also attempting to treat anorexia nervosa by inhibiting healthy individuals’ feces (Wilson et al., 2023). Furthermore, a recent meta-analysis also indicates variations in the microbiota of individuals with mental disorders (Nikolova et al., 2021). This study incorporated 59 case-control studies, including 10 related to AN (involving 211 patients). It revealed alterations in 12 microbial taxa among AN, though the results pertaining to these taxa were not consistent across all studies (Nikolova et al., 2021). Current research faces challenges in obtaining consistent conclusions by experimental design that can eliminate the influence of confounding factors. The MR method ingeniously mitigates the impact of confounding factors, thereby yielding consistent results (Emdin et al., 2017).

To our knowledge, this is the first MR study to ascertain the relationship between AN and gut microbiome. We found that there was genetic liability to 18 gut microbiotas causally associated with AN. Our study also found some results were consistent with previously researches. For instance, a high abundance of *Genus Lactobacillus*, in our study, is a risk factor for AN. Interestingly, a recent study also observed that the abundance of several *Lactobacillus* species was increased in the Activity-Based Anorexia mouse model (Breton et al., 2021). In another study, an increased abundance of *Class Actinobacteria* was observed in patients of AN (Nikolova et al., 2021). Interestingly, a similar increase in *Class Actinobacteria* abundance was also found in a clinical trial involving obese patients, coinciding with weight reduction in overweight individuals (Chambers et al., 2019).

However, our study also deviated from several previous research findings. Another study highlighted variations in the composition of fecal microbiota between AN patients upon admission and at discharge, specifically involving *Genus Anaerostipes* and *Genus Christensenellaceaer.7Group*. In their study, the abundance of these two taxa decreased from admission to discharge. Our study, in contrast, revealed that the growth of *Genus Anaerostipes* and *Genus Christensenellaceaer.7Group* was likely inhibited under AN condition. We hypothesized that this increase might stem from complex interactions within the microbial communities. In addition, *Family Coriobacteriaceae ID:811*was reported increasing in the patients of AN. However, AN, in our study, acted as an inhibitory factor for the growth of *Family Coriobacteriaceae ID:811*. *Family Coriobacteriaceae ID:811*, should be decease under the condition of the AN. It’s worth noting that our study also provided a clear conclusion for the conflicting results from prior research. For instance, a meta-analysis published in 2020 indicated a potential significant association between *Anaerostipes* and BMI (Di Lodovico et al., 2021). However, with an increase in studies, a meta-analysis in 2021 suggested inconsistent findings (Nikolova et al., 2021). In our study, we observed that *Genus Anaerostipes ID:1991* exhibited suppressed growth under condition of AN. One possible explanation for this difference could be that, while MR analysis eliminates the interference of various confounding factors, the inherent differences in the physical properties of the selected microbial samples themselves cannot be ignored. These differences may have led to variations in our study’s conclusions compared to previous findings. Standardized sample selection and large-scale randomized controlled trials can help eliminate the potential interference caused by these differences.

Meanwhile, our study has several limitations. Firstly, constrained by GWAS data, we can accurately examine the relationship between the genus level or higher taxonomic levels and AN, but cannot extend the validation to more specific levels, such as species. Secondly, due to the utilization of publicly available GWAS data, there was an imbalance in the number of case and control groups within these cohorts, potentially increasing the likelihood of pleiotropy. Concurrently, the impact of genetic mutations on other pathways might also impact the interpretability of the results. Thirdly, the GWAS data we utilized are derived from individuals of European ancestry. Therefore, it remains uncertain whether our conclusions are applicable to other populations, such as East Asian or South Asian populations. Fourthly, since we were unable to obtain individual-level gender data from the GWAS data, we were unable to analyze AN patients of different genders and, therefore, explore the interaction between microbial taxa and AN patients of different genders. Lastly, the absence of pertinent GWAS data precluded us from conducting validation through replication samples.

In summary, our analysis delved into the causal connection between 211 gut microbiota and AN. For the first time, we have not only showed that 10 gut microbiotas acted as risk or protective factors for AN, but also revealed that 8 gut microbiotas were influenced under AN condition. This study offered novel insights into the causal relationship between AN and gut microbial taxa.

## Data availability statement

The raw data supporting the conclusions of this article will be made available by the authors, without undue reservation.

## Author contributions

XX: Data curation, Formal analysis, Funding acquisition, Investigation, Writing – original draft. S-yH: Data curation, Formal analysis, Writing – original draft. X-LZ: Methodology, Software, Supervision, Writing – review & editing. DW: Funding acquisition, Methodology, Software, Supervision, Validation, Writing – review & editing. QH: Funding acquisition, Software, Validation, Writing – review & editing. Q-AX: Conceptualization, Data curation, Formal analysis, Investigation, Methodology, Resources, Writing – original draft. YY: Conceptualization, Funding acquisition, Project administration, Supervision, Validation, Visualization, Writing – review & editing.

## Abbreviations

AN: Anorexia nervosa
CI: Confidence interval
GCAN: Genetic consortium for anorexia nervosa
GWAS: Genome-wide association study
IV: Instrumental variables
IVW: Inverse variance weighted
LD: Linkage disequilibrium
MR: Mendelian randomization
MR-PRESSO: Mendelian randomization pleiotropy RESidual sum and outlier
mbQTL: Microbiota quantitative trait loci
OR: Odds ratio
RCT: Randomized control trial
SNPs: Single nucleotide polymorphisms
WM: Weighted median
WTCCC3: Wellcome trust case control consortium 3
