# Supplementary material for: The causality between gut microbiome and anorexia nervosa: a Mendelian randomization analysis
Source: Front Microbiol. 2023 Oct 19;14:1290246. doi: 10.3389/fmicb.2023.1290246 (PMC10620704; doi:10.3389/fmicb.2023.1290246)
Supplement: Supplementary file 1 [file Table_1.DOCX]

**Table 1: STROBE-MR checklist of recommended items to address in reports of Mendelian randomization studies**

| **Item No.** | **Section** | **Checklist item** | **Page No.** | **Relevant text from manuscript** |
| --- | --- | --- | --- | --- |
| 1 | **TITLE and ABSTRACT** | Indicate Mendelian randomization (MR) as the study’s design in the title and/or the abstract if that is a main purpose of the study | 1 | The causality between gut microbiome and anorexia nervosa: a Mendelian randomization analysis |
|  | **INTRODUCTION** |  |  |  |
| 2 | **Background** | Explain the scientific background and rationale for the reported study. What is the exposure? Is a potential causal relationship between exposure and outcome plausible? Justify why MR is a helpful method to address the study question | 5 | However, the specific role played by individual microbial groups in this process remains unclear. Recently, the crucial role of the gut microbiome in AN has been gradually unveiled. Nevertheless, exploring the connection between AN and the gut microbiota poses challenges owing to the susceptibility to various influencing factors….. |
| 3 | **Objectives** | State specific objectives clearly, including pre-specified causal hypotheses (if any). State that MR is a method that, under specific assumptions, intends to estimate causal effects | 6 | Mendelian randomization (MR) is a genetic epidemiological approach that utilizes instrumental variables (IVs) which are highly correlated with the exposure of interest, to investigate causality and mitigate the impact of confounding factors…… |
|  | **METHODS** |  |  |  |
| 4 | **Study design and data sources** | Present key elements of the study design early in the article. Consider including a table listing sources of data for all phases of the study. For each data source contributing to the analysis, describe the following: |  |  |
|  | a) | Setting: Describe the study design and the underlying population, if possible. Describe the setting, locations, and relevant dates, including periods of recruitment, exposure, follow-up, and data collection, when available. | 7 | The GWAS summary dataset encompassed 2,907 individuals diagnosed with AN across 14 countries, along with 14,860 ancestrally matched control subjects, constituting a part of the Genetic Consortium for Anorexia Nervosa (GCAN) and the Wellcome Trust Case Control Consortium 3 (WTCCC3) |
|  | b) | Participants: Give the eligibility criteria, and the sources and methods of selection of participants. Report the sample size, and whether any power or sample size calculations were carried out prior to the main analysis | 7 | The GWAS summary dataset encompassed 2,907 individuals diagnosed with AN across 14 countries, along with 14,860 ancestrally matched control subjects, constituting a part of the Genetic Consortium for Anorexia Nervosa (GCAN) and the Wellcome Trust Case Control Consortium 3 (WTCCC3) |
|  | c) | Describe measurement, quality control and selection of genetic variants | 7-8 | The criterion of selecting IVs as following: (1) SNPs, significantly associated with gut microbiota, were selected (the P value of SNPs < 1*10^-5^) as the potential eligible IVs…… |
|  | d) | For each exposure, outcome, and other relevant variables, describe methods of assessment and diagnostic criteria for diseases | 7 | All individuals were of European ancestry and detailed information of AN was available in the website (https://gwas. mrcieu.ac.uk/datasets/ieu-a-45/). |
|  | e) | Provide details of ethics committee approval and participant informed consent, if relevant | No applicable |  |
| 5 | **Assumptions** | Explicitly state the three core IV assumptions for the main analysis (relevance, independence and exclusion restriction) as well assumptions for any additional or sensitivity analysis | 6,8-9 | All MR analysis of this study was executed under three basic assumptions……  Five methods (including Inverse variance weighted (IVW), MR Egger, Weighted median, Simple mode, and Weighted mode method) were used to estimate the causal effect of the gut microbiota on AN. |
| 6 | **Statistical methods: main analysis** | Describe statistical methods and statistics used | 9 | A significance threshold for multiple testing was established at each taxonomic level (phylum, class, order, family, and genus). Bonferroni correction method was employed to adjust the P-values, mitigating the potential for false positives (P < 0.05/N, N refers to the effective number of independent bacterial taxa at the specific taxonomic level). |
|  | a) | Describe how quantitative variables were handled in the analyses (i.e., scale, units, model) | 7 | Five methods (including Inverse variance weighted (IVW), MR Egger, Weighted median, Simple mode, and Weighted mode method) were used to estimate the causal effect of the gut microbiota on AN. |
|  | b) | Describe how genetic variants were handled in the analyses and, if applicable, how their weights were selected | 7-8 | The criterion of selecting IVs as following: (1) SNPs, significantly associated with gut microbiota, were selected (the P value of SNPs < 1*10-5) as the potential eligible IVs |
|  | c) | Describe the MR estimator (e.g. two-stage least squares, Wald ratio) and related statistics. Detail the included covariates and, in case of two-sample MR, whether the same covariate set was used for adjustment in the two samples |  | The list of covariates varies between original GWASs, but always included sex and age. Details can be found in the original studies. |
|  | d) | Explain how missing data were addressed | No applicate |  |
|  | e) | If applicable, indicate how multiple testing was addressed |  | Bonferroni correction method was employed to adjust the P-values, mitigating the potential for false positives (P < 0.05/N, N refers to the effective number of independent bacterial taxa at the specific taxonomic level). The significant P values were following:0.00038(131 Genera), 0.0014(35 Families), 0.0025(20 Orders), 0.0031(16 Classes), and 0.0056 (9 Phyla). |
| 7 | **Assessment of assumptions** | Describe any methods or prior knowledge used to assess the assumptions or justify their validity | 8 | It's worth noting that, while the IVW method assumes the validity of all instrumental variables, it can be influenced by instrumental variable pleiotropy and heterogeneity. |
| 8 | **Sensitivity analyses and additional analyses** | Describe any sensitivity analyses or additional analyses performed (e.g. comparison of effect estimates from different approaches, independent replication, bias analytic techniques, validation of instruments, simulations) | 9 | The significant microbiotas were tested for pleiotropy and heterogeneity to ensure the accuracy of the IVW results. MR-Egger Intercept Test and Mendelian Randomization Pleiotropy RESidual Sum and Outlier (MR-PRESSO) global test were employed to detect horizontal pleiotropy |
| 9 | **Software and pre-registration** |  |  |  |
|  | a) | Name statistical software and package(s), including version and settings used |  | All data processing and analysis were accomplished by R software (R.4.2.3; http://www.R-project.org). The R packages used in study is TwoSampleMR, MendelianRandomization, and MR-PRESSO. |
|  | b) | State whether the study protocol and details were pre-registered (as well as when and where) | No Applicable |  |
|  | **RESULTS** |  |  |  |
| 10 | **Descriptive data** |  |  |  |
|  | a) | Report the numbers of individuals at each stage of included studies and reasons for exclusion. Consider use of a flow diagram | Figure 1 | the detail information displayed in Figure 1 |
|  | b) | Report summary statistics for phenotypic exposure(s), outcome(s), and other relevant variables (e.g. means, SDs, proportions) | 7 | In the original study, the gut microbiota was categorized into 257 taxa at six taxonomic levels: Phylum, Class, Order, Family, and Genus. Finally, 211 taxa were defined, including 131 genera, 35 families, 20 orders, 16 classes, and 9 phyla. |
|  | c) | If the data sources include meta-analyses of previous studies, provide the assessments of heterogeneity across these studies | No Applicable |  |
|  | d) | For two-sample MR:  i.  Provide justification of the similarity of the genetic variant-exposure associations between the exposure and outcome samples  ii.  Provide information on the number of individuals who overlap between the exposure and outcome studies | 7 | The GWAS data we selected all originate from populations of European ancestry. These individuals are largely independent of each other. |
| 11 | **Main results** |  |  |  |
|  | a) | Report the associations between genetic variant and exposure, and between genetic variant and outcome, preferably on an interpretable scale | 11-12 | Under the condition of IVW < 0.05, 10 gut microbiotas were associated with AN (Figure 2, 4A)…… |
|  | b) | Report MR estimates of the relationship between exposure and outcome, and the measures of uncertainty from the MR analysis, on an interpretable scale, such as odds ratio or relative risk per SD difference | No Applicate |  |
|  | c) | If relevant, consider translating estimates of relative risk into absolute risk for a meaningful time period | No Applicable |  |
|  | d) | Consider plots to visualize results (e.g. forest plot, scatterplot of associations between genetic variants and outcome versus between genetic variants and exposure) | Figure 2,3,4 | Under the condition of IVW < 0.05, 10 gut microbiotas were associated with AN (Figure 2, 4A). |
| 12 | **Assessment of assumptions** |  |  |  |
|  | a) | Report the assessment of the validity of the assumptions | 11 | MR PRESSO didn’t identify heterogeneity among the significant and potential microbiota (Table 1, 2). |
|  | b) | Report any additional statistics (e.g., assessments of heterogeneity across genetic variants, such as *I^2^*, Q statistic or E-value) | 11 | Similarly, Cochran's Q test indicated the absence of heterogeneity across the studies (Table 1, 2). |
| 13 | **Sensitivity analyses and additional analyses** |  |  |  |
|  | a) | Report any sensitivity analyses to assess the robustness of the main results to violations of the assumptions | 11 | MR PRESSO didn’t identify heterogeneity among the significant and potential microbiota (Table 1, 2). Similarly, Cochran's Q test indicated the absence of heterogeneity across the studies (Table 1, 2). Moreover, the Leave-one-out analysis revealed no significant differences in SNP effects. Furthermore, the MR Egger regression did not yield any evidence of horizontal pleiotropy (*P* > 0.05), and all F-statistical values exceeded 10 (Table 1, 2). |
|  | b) | Report results from other sensitivity analyses or additional analyses | No applicate |  |
|  | c) | Report any assessment of direction of causal relationship (e.g., bidirectional MR) | 11 | In the reverse MR, eight gut microbial taxa were identified that are correlated with AN |
|  | d) | When relevant, report and compare with estimates from non-MR analyses | No applicate |  |
|  | e) | Consider additional plots to visualize results (e.g., leave-one-out analyses) | 11, Figure 5 | The MR analysis and Leave-one out analysis result of *Class Actinobacteria ID:419* was shown in the Figure 5. |
|  | **DISCUSSION** |  |  |  |
| 14 | **Key results** | Summarize key results with reference to study objectives | 13 | To our knowledge, this is the first MR study to ascertain the relationship between AN and gut microbiome. We found that there was genetic liability to 18 gut microbiotas causally associated with AN |
| 15 | **Limitations** | Discuss limitations of the study, taking into account the validity of the IV assumptions, other sources of potential bias, and imprecision. Discuss both direction and magnitude of any potential bias and any efforts to address them | 14,15 | Meanwhile, our study has several limitations. Firstly, constrained by GWAS data, we can accurately examine the relationship between the genus level or higher taxonomic levels and AN…… |
| 16 | **Interpretation** |  |  |  |
|  | a) | Meaning: Give a cautious overall interpretation of results in the context of their limitations and in comparison with other studies | 15 | For the first time, we have not only showed that 10 gut microbiotas acted as risk or protective factors for AN, but also revealed that 8 gut microbiotas were influenced under AN condition. |
|  | b) | Mechanism: Discuss underlying biological mechanisms that could drive a potential causal relationship between the investigated exposure and the outcome, and whether the gene-environment equivalence assumption is reasonable. Use causal language carefully, clarifying that IV estimates may provide causal effects only under certain assumptions | No applicate |  |
|  | c) | Clinical relevance: Discuss whether the results have clinical or public policy relevance, and to what extent they inform effect sizes of possible interventions | 15 | This study offered novel insights into the causal relationship between AN and gut microbial taxa. |
| 17 | **Generalizability** | Discuss the generalizability of the study results (a) to other populations, (b) across other exposure periods/timings, and (c) across other levels of exposure | 15 | Thirdly, the GWAS data we utilized are derived from individuals of European ancestry. Therefore, it remains uncertain whether our conclusions are applicable to other populations, such as East Asian or South Asian populations. |
|  | **OTHER INFORMATION** |  |  |  |
| 18 | **Funding** | Describe sources of funding and the role of funders in the present study and, if applicable, sources of funding for the databases and original study or studies on which the present study is based | 15-16 | This work was supported by Open Foundation of Hubei Key Laboratory of Tumor Microenvironment and Immunotherapy，China Three Gorges University (2023KZL012), National Natural Science Foundation of China (No. 31772709, 82100473) and Hubei Natural Science Foundation (2015CFB316) of China. |
| 19 | **Data and data sharing** | Provide the data used to perform all analyses or report where and how the data can be accessed, and reference these sources in the article. Provide the statistical code needed to reproduce the results in the article, or report whether the code is publicly accessible and if so, where | 15 | The GWAS data of gut microbiome were obtained from https://mibiogen. gcc.rug.nl/. The GWAS data of anorexia nervosa was acquired from the IEU open GWAS project (https://gwas.mrcieu.ac.uk/), which mainly refers from the Genetic Consortium for AN (GCAN). |
| 20 | **Conflicts of Interest** | All authors should declare all potential conflicts of interest | 15 | All authors read and approved the final manuscript. |

This checklist is copyrighted by the Equator Network under the Creative Commons Attribution 3.0 Unported (CC BY 3.0) license.

1. Skrivankova VW, Richmond RC, Woolf BAR, Yarmolinsky J, Davies NM, Swanson SA, et al. Strengthening the Reporting of Observational Studies in Epidemiology using Mendelian Randomization (STROBE-MR) Statement. JAMA. 2021; under review.

2. Skrivankova VW, Richmond RC, Woolf BAR, Davies NM, Swanson SA, VanderWeele TJ, et al. Strengthening the Reporting of Observational Studies in Epidemiology using Mendelian Randomisation (STROBE-MR): Explanation and Elaboration. BMJ. 2021;375: n2233.
