# Supplementary figures and images for: The causality between gut microbiome and anorexia nervosa: a Mendelian randomization analysis
Source: Front Microbiol. 2023 Oct 19;14:1290246. doi: 10.3389/fmicb.2023.1290246 (PMC10620704; doi:10.3389/fmicb.2023.1290246)

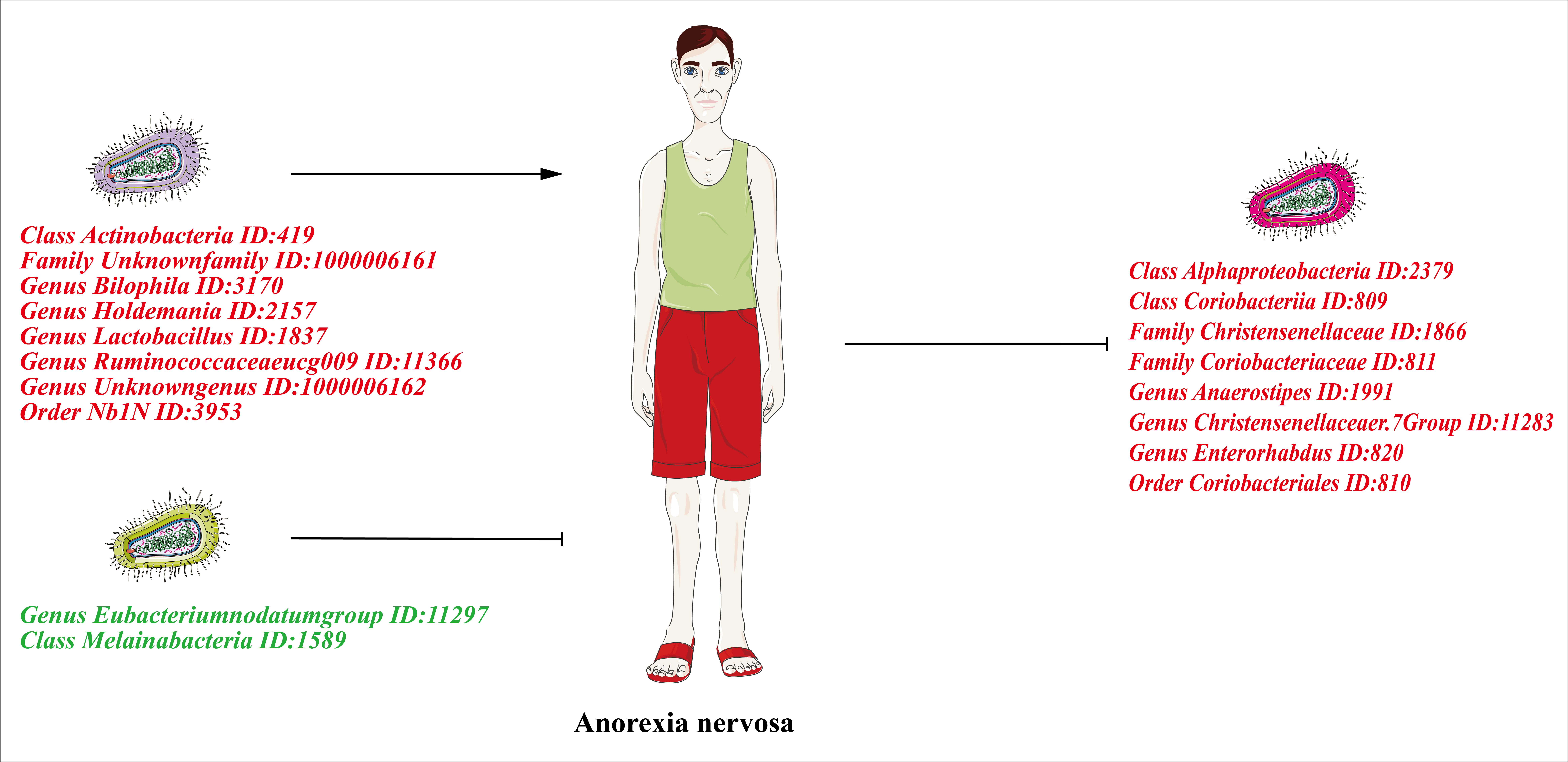

Supplement: Supplementary file 4 [file Image_1.JPEG]
